# The characteristics of cortical activation during gait in children with spastic diplegic cerebral palsy

**DOI:** 10.3389/fped.2026.1661105

**Published:** 2026-03-12

**Authors:** Jin Wang, Jinmei Zhu, Haiying Zhu, Chuan Guo, Tong Wang, Jijiang Zhou, Jun He, Shizhe Zhu, Tongbo Lu

**Affiliations:** 1 Department of Rehabilitation Medicine, Changzhou Dean Hospital, Changzhou, China; 2 School of Medicine, Jiangsu University, Zhenjiang, China; 3 Department of Rehabilitation Medicine, The First Affiliated Hospital of Nanjing Medical University, Nanjing, China

**Keywords:** cerebral palsy, cortical activation, CTD, fNIRS, gait, spastic DCP

## Abstract

**Introduction:**

Cerebral palsy (CP) is a common movement disorder caused by abnormalities or injury to the developing brain. It affects more than 17 million people worldwide and is associated with substantial impairments in postural balance and gait control, particularly in children with spastic diplegic CP. This study aimed to examine differences in cortical activation during walking between children with spastic diplegic CP and children with typical development (CTD), and to explore the relationship between cortical activation and gross motor performance.

**Methods:**

Functional near-infrared spectroscopy (fNIRS) was used to monitor cortical activity during walking in 15 children with spastic diplegic CP and 15 age-matched CTD participants. All participants walked at a self-selected pace while cortical hemodynamic responses were recorded. Cortical activation patterns were compared between groups, and correlation analyses were conducted to evaluate associations between cortical activation, gross motor function, and walking speed.

**Results:**

Compared with CTD, children with spastic diplegic CP showed significantly greater cortical activation in the right prefrontal cortex (RPFC), left prefrontal cortex (LPFC), and right premotor cortex (RPMC) during walking. In addition, activation in the RPMC was negatively correlated with gross motor function and walking speed.

**Discussion:**

These findings indicate that children with spastic diplegic CP exhibit cortical over-activation during walking, particularly in the prefrontal and premotor cortices. The observed negative association between RPMC activation and motor performance suggests that greater cortical recruitment may reflect increased compensatory demands during motor planning and gait control. Overall, the results support the notion that children with spastic diplegic CP rely more heavily on cortical compensatory mechanisms to maintain walking performance.

## 1 Introduction

Cerebral palsy (CP) is a movement disorder caused by an abnormality or injury to the developing brain (1). It is the primary cause of movement disorders in children (2) and has emerged as a significant public health concern, affecting over 17 million people globally (3). Spastic CP, the most common type of CP, gives rise to difficulties in posture, balance, and gait control (4). Children with CP can reach 90% of their gross motor potential by age 5, with the most potential achieved by 3 years (5). The potential of early motor intervention to optimize functional outcomes by harnessing the greater neuroplasticity of the developing brain and neuromotor system is particularly relevant to CP (6). A study (7) demonstrates that robot-assisted gait training can induce detectable cortical functional plasticity in children with CP, as reflected by modulation of prefrontal and sensorimotor cortical activation associated with improvements in motor function. Furthermore, fNIRS-derived measures may serve as potential neurobiological markers for evaluating and predicting rehabilitation outcomes. Sterling et al.'s study (8) showed that after 10 individuals with unilateral CP received constraint-induced movement therapy, the volume of grey matter in the sensorimotor cortex increased. Understanding the brain characteristics of CP and identifying the differences between spastic diplegia CP and children with typical development (CTD) will help enhance functional recovery in the future.

Previous brain imaging studies have often utilized functional magnetic resonance imaging (fMRI), diffusion tensor imaging (DTI), and electroencephalography (EEG). In individuals with spastic diplegic CP, reduced fractional anisotropy (FA) values were identified in several white matter regions, including bilateral white matter tracts in the prefrontal and temporal lobes, as well as the internal and external capsules, using DTI (9). Furthermore, a significant association was found between damage to several of these white matter tracts and motor dysfunction (10). A distinct pattern in resting-state regional brain activities and functional connectivity (FC) was revealed in patients with occult spastic diplegic CP compared to a control group. These findings were not limited to resting-state observations, as task-related states were also examined. Specifically, fMRI studies have demonstrated that in children with hemiplegic CP, the unaffected hemispheres often compensate for the damaged hemispheres during tasks such as finger-to-thumb opposition (11, 12). However, at the level of activity and participation as defined by the International Classification of Functioning, Disability and Health framework, walking and functional mobility represent core domains of everyday functioning that are commonly affected in children with spastic diplegic cerebral palsy. While impairments in lower limb–related activities have been investigated using behavioral and clinical assessments, evidence at the level of body functions—particularly studies integrating brain imaging during task-related lower limb activities—remains limited in this population. This limitation is largely attributable to earlier neuroimaging techniques being unable to support real-time monitoring during dynamic lower limb movements, which restricted investigations primarily to resting-state paradigms or upper limb tasks.

Among the currently available neuroimaging tools, functional near-infrared spectroscopy (fNIRS) stands out as the most appropriate technique for assessing trials that involve extensive locomotion. fNIRS uses near-infrared light, enabling the detection of oxyhemoglobin (Oxy-Hb) and deoxyhemoglobin (Deoxy-Hb) (13). The phenomenon of increased cortical activation, accompanied by a spontaneous rise in blood flow within the cortices, is referred to as neurovascular coupling (14). Changes in Oxy-Hb and Deoxy-Hb measured by fNIRS have been consistently shown to reflect task-related neural activity through neurovascular coupling (15). Compared to fMRI and EEG, fNIRS is less affected by motion artifacts and is better suited for complex motor activities like walking (16). Furthermore, Oxy-Hb has been reported to be more sensitive to changes in cortical activity associated with walking (17). As a result, the β value based on Oxy-Hb was selected as the indicator in this study to assess cortical activity. The β value, which is indicative of the level of cortical activation in a given channel, was employed as an estimator for predicting hemodynamic response functions of the HbO signal, representing the peak value of the hemodynamic response function (18). The testing procedures are also safe and feasible for children (19).

Although several research on children with cerebral palsy has been conducted, studies using brain imaging techniques to investigate brain network changes during lower-limb function remain limited. To date, only one study has explored cortical activation during walking in this population. Kurz et al.'s study (20) aimed to investigate differences in cortical activation during gait between children with spastic diplegic CP and CTD. Using fNIRS, increased activation in the sensorimotor cortices and superior parietal lobule was found in children with spastic diplegic CP, along with greater variability in stride time intervals. However, Kurz et al.'s study only analyzed four spastic diplegic CP and eight CTD and the small sample size could result in limited reliability of the evidence. At the same time, they required all participants to walk on the treadmill at the same speed. Spastic diplegic CP often have a slower average walking speed compared to CTD (21). Controlling both groups to walk at the same speed may affect the recruitment of peripheral muscles and neural activation (22). Individual differences may have a significant impact on the results. Another study by (16) focused on lower extremity motor tasks. The results showed that, among children with CP classified as GMFCS levels I–III, they showed more cortical activation compared with CTD and greater limitations in movement were associated with higher levels of sensorimotor cortical activation and increased muscle co-activation. These findings correlated with decreased motor selectivity and functional mobility, suggesting a neural mechanism underlying motor limitation and potential targets for intervention.

Previous studies have demonstrated that the prefrontal cortex, premotor cortex (PMC), sensorimotor cortex (SMC), and visual cortex (VC) are all involved in the cortical control of self-selected walking pace (23). This approach also aims to monitor cortical activation under conditions that reflect daily walking state with a larger simple size. Based on previous research, it is hypothesized that individuals with spastic diplegic CP will exhibit more significant bilateral activation in the SMC and PFC during walking compared to CTD. Additionally, it is hypothesized that the activation of the SMC and PFC is significantly correlated with walking speed.

## 2 Method

### 2.1 Participants

A total of 15 participants with spastic diplegic CP and 15 CTD were ultimately included in the study from 1st March 2022 to 31st December 2023. Detailed participant information is provided in Table 1. The inclusion criteria for spastic CP were as follows: (1) children aged 6–16 years with a confirmed diagnosis of spastic diplegic CP, defined as a group of permanent disorders of movement and posture caused by non-progressive disturbances to the developing fetal or infant brain by a pediatric neurologist. The diagnosis was based on clinical history, neurological examination, and neuroimaging findings when available, and was characterized by predominant spastic involvement of the bilateral lower extremities, with relatively greater impairment in the lower limbs than the upper limbs; (2) Gross Motor Function Classification System Level I-II with verbal supervision and protective support during walking; and (3) required to demonstrate the ability to understand and follow simple verbal instructions during a brief familiarization session prior to data collection. The exclusion criteria were: (1) a history of brain or selective dorsal rhizotomy surgery; (2) botulinum toxin injections and use of Baclofen within the last six months; (3) the level of Modified Ashworth Scale > 2 (hip adductors, knee flexors, and ankle plantar flexors); and (4) children with additional orthopedic, cardiopulmonary, or peripheral nerve conditions that were unrelated to cerebral palsy and were deemed to significantly affect gait were excluded. Additional orthopedic conditions indicated recent lower limb surgery within the past year or acute orthopedic conditions, such as fracture and sprain, that were deemed unrelated to their CP diagnosis and likely to significantly affect gait performance. Peripheral neuropathy was screened based on a combination of detailed medical history review and standardized neurological examination conducted by experienced clinicians. Specifically, participants were assessed for clinical signs suggestive of peripheral neuropathy, including distal sensory loss, asymmetric sensory deficits, abnormal reflex patterns inconsistent with upper motor neuron involvement, and a history of conditions known to cause peripheral nerve damage (e.g., metabolic disease, trauma, or neurotoxic exposure). For the CTD group, participants were matched primarily by age and had the same exclusion criteria. Informed consent was provided by participants’ guardians and assent was achieved by these children at the same time. The study was approved by the Dean Ethics Committee in Changzhou (approval number: CZDALL-2021-006). The studies were conducted in accordance with the local legislation and institutional requirements. After signing the consent, all participants were assessed using the Gross Motor Function Measure (GMFM) D part (including sitting) and E part (including walking, running, and jumping), Pediatric Balance Scale (PBS), and 10 m gait speed. All scales were conducted by a licensed physical therapist who had received standardized training in these instruments.

**Table 1:** Characteristics of participants.

Characteristics	CP (*n* = 15)	CTD (*n* = 15)
Sex (female, %)	8 (57.1%)	8 (57.1%)
Age (Mean ± SD)	12.53 ± 2.73	13.00 ± 3.34
GMFM-D (Mean ± SD)	25.37 ± 6.03	39.00 ± 0.00
GMFM-E (Mean ± SD)	42.80 ± 13.33	72.00 ± 0.00
PBS (Mean ± SD)	32.53 ± 8.35	55.87 ± 0.35
10 m walking speed (m/s) (Mean ± SD)	0.59 ± 0.21	1.36 ± 0.14

CP, cerebral palsy; CTD, children with typical development; GMFM, gross motor function Measure; PBS, pediatric balance scale.

### 2.2 Experimental procedure

**Figure 1:**
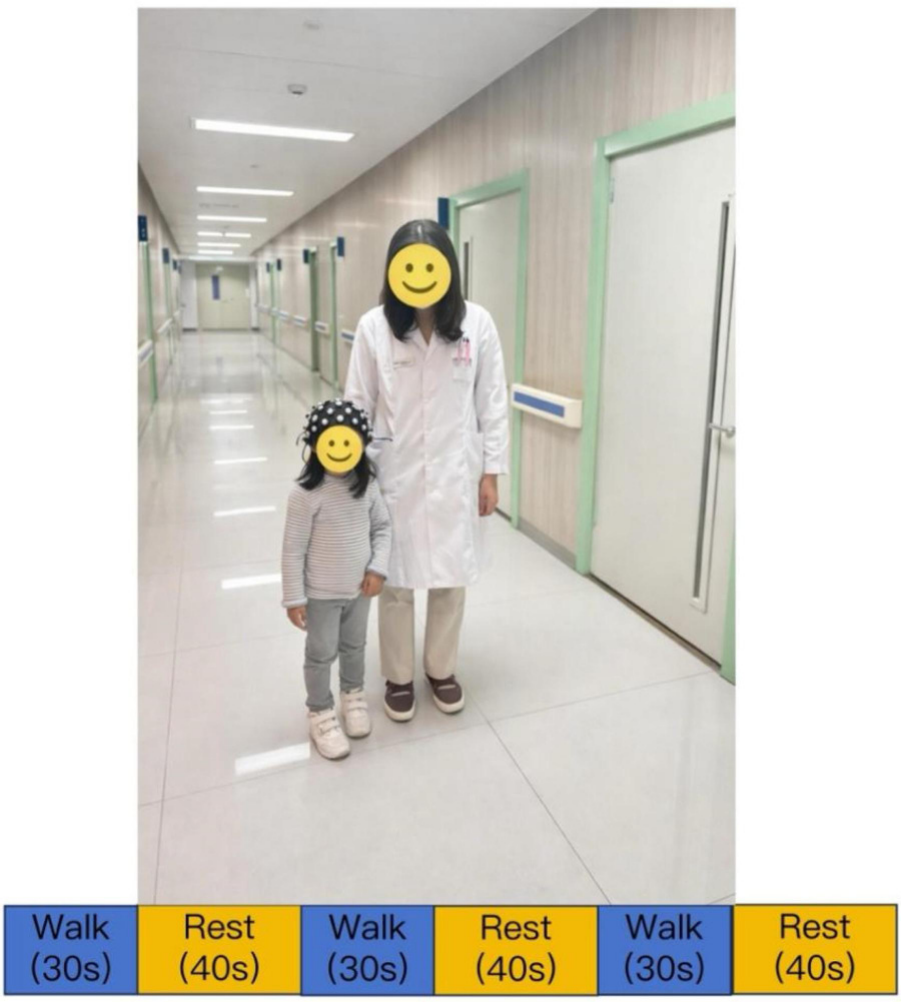
Illustration of fNIRS cap placement and the experimental block design.

After evaluations of all scales, participants were asked to complete a walking task while wearing fNIRS equipment for real-time monitoring. The walking task was designed using a block design, consisting of 30 s of walking followed by 40 s of rest (quiet standing), repeated three times (Figure 1). Participants walked at a self-selected walking pace. All participants performed the walking task wearing their own shoes, and no orthotic devices were permitted during testing. All assessments were conducted in a hospital setting, and the walking trials were performed on a level tiled floor. To reduce unfamiliarity and fear of the fNIRS equipment, all participants underwent 1–2 practice trials before the formal experiment, with a 10-minute rest period between the practice and the actual test.

### 2.3 fNIRS measurement

**Figure 2:**
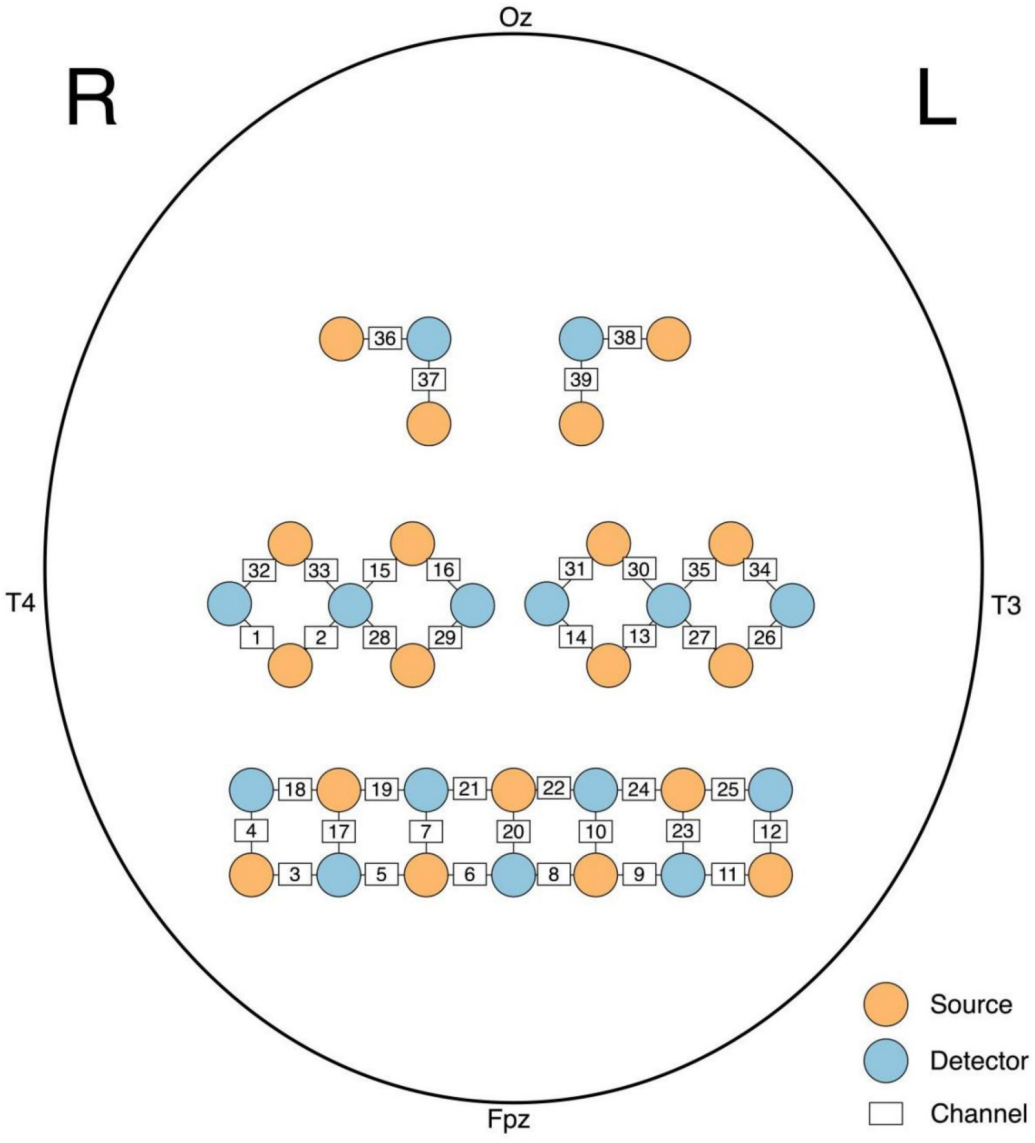
The attribution of sources, detectors and channels on the standard headform. R-, right, L-, left, Fpz, frontopolar midline electrode; Oz, Occipital midline electrode; T3, left temporal electrode, T4, right temporal electrode.

The NirSmart fNIRS device (Danyang Huichuang Medical Equipment Co., Ltd., Zhenjiang, China) was used in this study. The frequencies of near-infrared light were 730 and 850 nm, with an 11 Hz sampling frequency. Twenty source optodes and 15 detector optodes were combined into 39 channels to monitor the cortex (Figure 2). An electromagnetic 3D digitizer device (Patriot, Polhemus, Colchester, VT, USA) was utilized to determine the spatial information. The channels were then registered to the Montreal Neurological Institute (MNI) space and projected onto the MNI brain template. With the specific spatial coordinates, the percentage of coverage of various Brodmann's areas was obtained. Areas with the highest percentage were considered as the representative functional areas. More details of the division of regions of interest (ROI) are shown in Table 2.

**Table 2:** Division of the ROIs.

Brodmann area	Channel
RPFC	5, 6, 7, 17, 19, 21
LPFC	8, 9, 19, 22, 24
RPMC	1, 2, 15, 16, 28, 29
LPMC	13, 14, 26, 27, 30, 31
RSMC	32, 33
LSMC	34, 35
RVC	36, 37
LVC	38, 39

RPFC, right prefrontal cortex; LPFC, left prefrontal cortex; RPMC, right pre-motor cortex; LPMC, left pre-motor cortex; RSMC, right sensorimotor cortex; LSMC, left sensorimotor cortex; RVC, right visual cortex; LVC, left visual cortex.

### 2.4 fNIRS data analysis

The NirSpark software (HuiChuang, Zhenjiang, China) was used in our study to analyze the fNIRS data. First, we took several preprocessing steps: (1) The raw signals were transformed into the optical density signal; (2) We used the cubic spline interpolation algorithm (STDEV threshold = 6.0, AMP threshold = 0.5) to remove motion artifacts; (3) The task-related signals were primarily distributed between 0.01 and 0.1 Hz. Therefore, we used filtering to retain signals within this range (24); (4) The modified Beer-Lambert law with the path length factor of 6 was applied to convert the optical density signal to blood oxygen concentration (25). ΔOxy-Hb was chosen as the primary indicator in our analysis due to its generally superior signal-to-noise ratio compared to Deoxy-Hb (26). Based on the Oxy-Hb signal, a generalized linear model was used to analyze the time series data. Each ROI's β value of all participants in the two groups was obtained. The walking task was repeated three times, and the final β-values for each participant were averaged over these three repetitions.

### 2.5 Statistical analysis

After obtaining the value of concentration changes, we used SPSS version 23.0 (IBM Corporation, Armonk, NY, USA) for further analysis. We used the Shapiro–Wilk test to examine whether the data followed a normal distribution. For most of the data not obeying the normal distribution and small simple size, we used the Mann–Whitney *U*-test to detect significance. We took the false discovery rate (FDR) correction (Benjamini-Hochberg correction). Results were considered statistically significant when the adjusted *p*-value (adj-p) was less than 0.05. Additionally, we performed a correlation analysis between the ROIs with significant differences and motor performance using the Spearman correlation coefficient.

## 3 Results

### 3.1 fNIRS results

**Figure 3:**
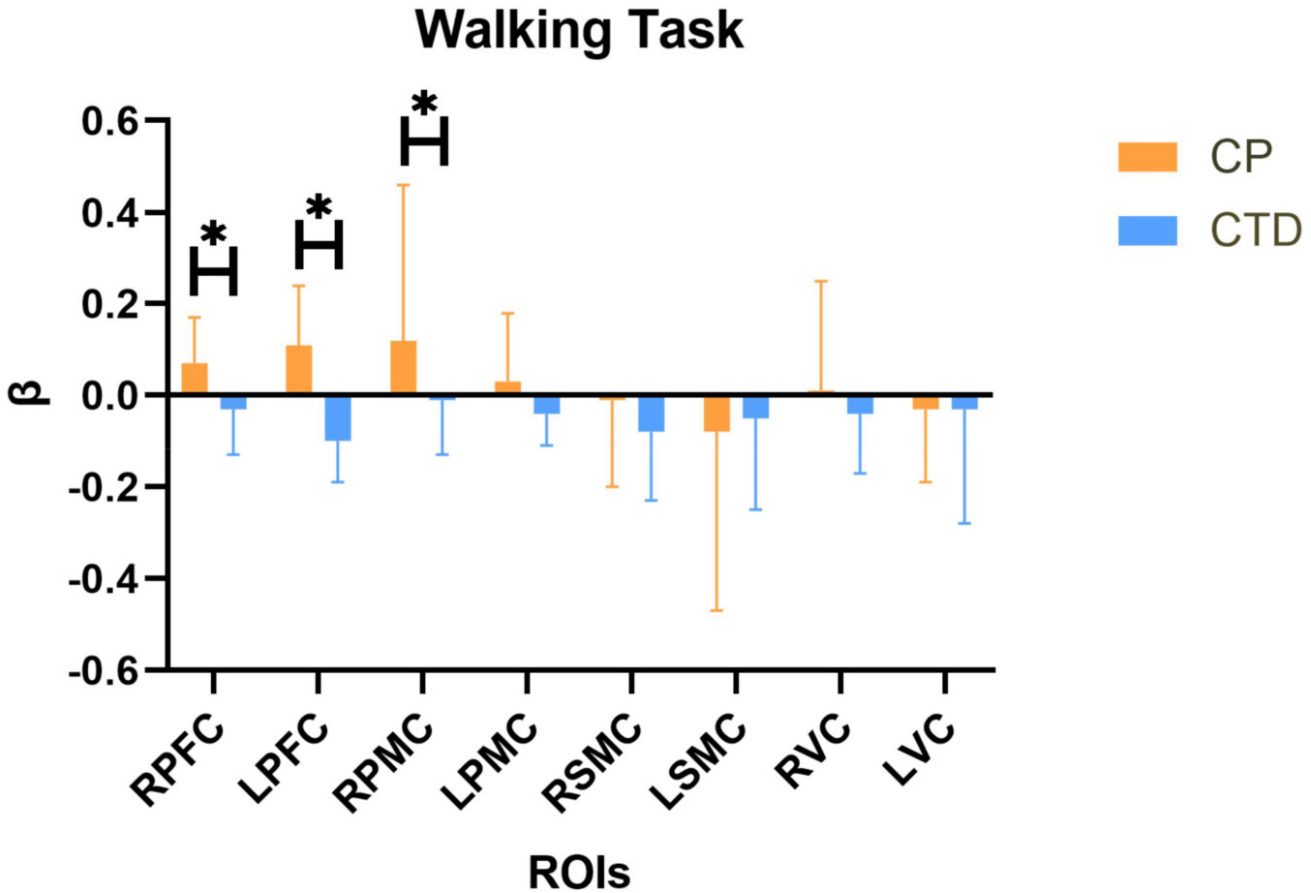
The comparision of β value beteween two groups in different ROIs. CP, cerebral palsy; CTD, children with typically development; RPFC, right prefrontal cortex; LPFC, left prefrontal cortex; RPMC, right pre-motor cortex; LPMC, left pre-motor cortex; RSMC, right sensorimotor cortex; LSMC, left sensorimotor cortex; RVC, right visual cortex; LVC, left visual cortex; *, adj-*p* < 0.05.

For the β value, significant statistical differences were observed between the children with spastic diplegic cerebral palsy group and the children with typical development group in several regions of interest. Specifically, a significant difference was found in the right prefrontal cortex between the two groups (Z = −2.84, adjusted *p* = 0.01). A significant difference was also observed in the left prefrontal cortex (Z = −4.58, adjusted *p* < 0.001). In addition, a significant difference was identified in the right premotor cortex (Z = −3.22, adjusted *p* = 0.01). No statistically significant differences were found in the left premotor cortex, right sensorimotor cortex, left sensorimotor cortex, right visual cortex, or left visual cortex. Details are presented in Table 3 and Figure 3.

**Table 3:** The Oxy-Hb of each ROIs.

ROI	CP（MD ± SD）	CTD（MD ± SD）	z	Adj-p
RPFC	0.07 ± 0.10	−0.03 ± 0.10	−2.84	0.01*
LPFC	0.11 ± 0.13	−0.10 ± 0.09	−4.58	<0.001*
RPMC	0.12 ± 0.34	−0.01 ± 0.12	−3.22	0.01*
LPMC	0.03 ± 0.15	−0.04 ± 0.07	−2.22	0.05
RSMC	−0.01 ± 0.19	−0.08 ± 0.15	−0.56	0.66
LSMC	−0.08 ± 0.39	−0.05 ± 0.20	−0.15	0.88
RVC	0.01 ± 0.24	−0.04 ± 0.13	−0.93	0.56
LVC	−0.03 ± 0.16	−0.03 ± 0.25	−0.64	0.66

CP, cerebral palsy; CTD, children with typical development; RPFC, right prefrontal cortex; LPFC, left prefrontal cortex; RPMC, right pre-motor cortex; LPMC, left pre-motor cortex; RSMC, right sensorimotor cortex; LSMC, left sensorimotor cortex; RVC, right visual cortex; LVC, left visual cortex.

*, adj-*p* < 0.05.

### 3.2 Correlations between cortical activation and motor function

**Figure 4:**
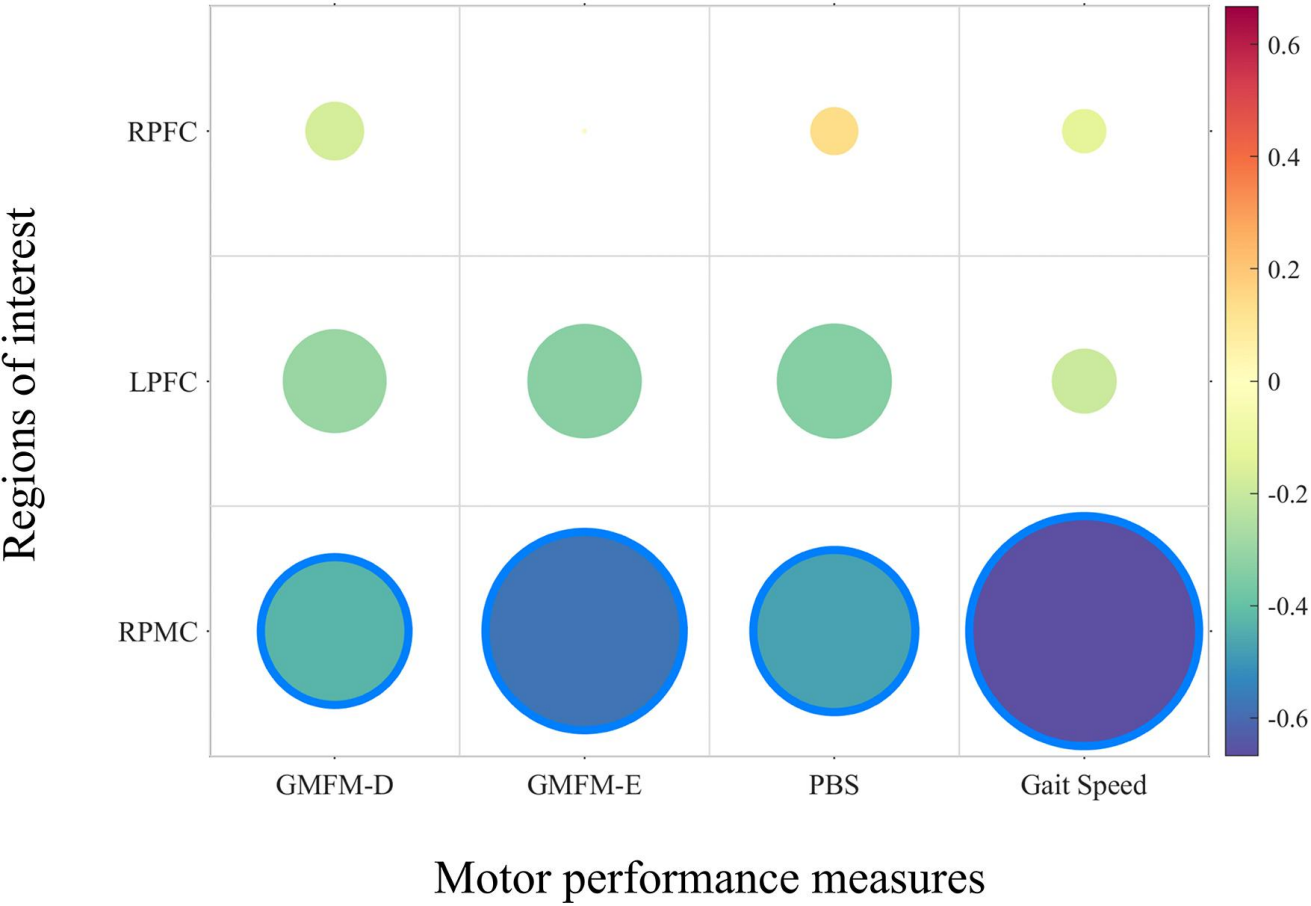
The correlation of the ROIs’ β values and motor performance. RPFC, right prefrontal cortex; LPFC, left prefrontal cortex; RPMC, right pre-motor cortex; GMFM, gross motor function Measure; PBS, pediatric balance scale.

Regions of interest that showed significant group differences also demonstrated significant associations with certain motor performance measures. Specifically, the β value of the right premotor cortex exhibited significant negative correlations with the Gross Motor Function Measure, Dimension E (*ρ* = −0.576, *p* = 0.025) and 10-meter walking speed (*ρ* = −0.668, *p* = 0.007). For the other regions of interest, no significant correlations were observed with the functional measures, as all *p*-values exceeded the 0.05 significance threshold. Details are presented in Figure 4.

## 4 Discussion

The primary aim of our study was to investigate the differences in cortical activation during walking between individuals with spastic diplegic CP and CTD and to explore the associations between cortical activation and peripheral behavioral performance. Higher activations were found for spastic diplegic CP in several brain regions, including the RPFC, LPFC, and RPMC. No significant differences were observed in the LPMC, RSMC, and LSMC. Additionally, significant correlations were found between the right RPMC and motor scales, indicating its relevance to motor function. No significant correlations were found for other brain regions.

Widespread cortical activation in individuals with cerebral palsy may result from the de-automation of walking. It is well established that the basic rhythm and patterning of locomotion are largely generated by subcortical and spinal mechanisms, including brainstem locomotor regions, spinal pattern-generating circuits, and cerebellar networks (27). At the same time, descending cortical pathways—particularly the corticospinal tract, which originates in the cerebral cortex—contribute to the modulation of gait even during automated walking by providing supervisory control over muscle activation, postural adjustments, and context-dependent adaptations. When automatic locomotor control is compromised, as in cerebral palsy, the reliance on cortical motor regions and corticospinal drive may increase to compensate for inefficient subcortical and spinal control, which may contribute to the widespread cortical activation observed during walking in this population. Disruption of these structures leads to a breakdown in the balance, resulting in additional resource consumption. This phenomenon can be observed in patients with neurological disorders such as CP, Parkinson's disease (PD), and stroke (20, 28, 29). Our results showed that in CTD, the activation of each ROI during walking was insufficient, aligning with the theory of gait automation, where subcortical neural structures dominate walking, and the cortex is minimally involved in the process. In contrast, individuals with spastic diplegic CP exhibited a significant increase in activation in both the PFC and the PMC.

The over-activation of the PFC during walking in individuals with CP may be a form of compensatory recruitment for the additional planning and execution of motor tasks. The PFC is associated with cognitive functions and also plays a significant role in motor activities. It is primarily involved in motor execution, including selective attention and inhibitory processes (30). The over-recruitment of the PFC during motor activities can be attributed to various neural mechanisms, such as inefficient processing, reactive recruitment due to limitations in task performance, and compensatory recruitment when effective recruitment is lacking (31). CP is often accompanied by more deviations from typical gait kinematics (20), with gait speed significantly slower than in CTD. Therefore, the increased activation of the PFC suggests a neural inefficiency to maintain motor performance in CP. This phenomenon of PFC over-activation has also been observed in upper limb trials, with Lee et al.'s study (32) finding that children with CP exhibited additional activation in the PFC, PMC, and superior parietal cortex during a hand-grasping task compared with CTD. Furthermore, in neurotypical individuals, increased activation of the PFC has been observed during precise stepping tasks that require more attentional control (33). PFC is more activated when the gait task requires high levels of attention. Similar patterns have been observed in individuals with stroke, in whom impaired gait control is associated with increased cortical activation during walking compared with neurotypical individuals (34).

An impairment in automatic movement and postural control may be a significant factor contributing to the increased activation of the PMC. The PMC includes the primary motor area (PMA) and supplementary motor area (SMA). The PMA is involved in sequencing movements activated by external stimuli, which is particularly relevant to the completion of gait adaptability tasks (35). Pelicioni et al.'s study observed that in patients with PD, there is increased activation of the PMA compared to neurotypical individuals, which is associated with reduced efficiency in motor control automaticity. The SMA has been proven to participate in balance control (36) and is crucial for coordinating rhythmic arm and leg movements between limbs (37). Increased activation of the SMA was observed during walking, requiring more balance control (36). Although we only found significant differences in the RPMC and LPMC also approached statistical significance. This lateralized pattern may indicate that premotor cortical engagement during walking is not strictly symmetric in individuals with CP. However, this interpretation should be viewed cautiously, as hemispheric differences in premotor recruitment have been reported in the context of increased motor planning and coordination demands (38). The reduced sensitivity of the test can be also attributed to the comparison of multiple brain regions and the application of multiple comparison corrections. When relating these findings to our results, the significant negative correlation between RPMC activation and both GMFM-D scores and 10-m walking speed suggests that individuals with poorer gross motor performance rely more heavily on premotor cortical resources during walking. Specifically, lower GMFM-D scores and slower walking speed were associated with greater recruitment of the right PMC, indicating increased demands on motor planning and control. This interpretation is consistent with previous reports showing that gait impairments in individuals with CP are characterized not only by reduced walking speed but also by increased gait variability (20) and difficulties in interlimb reciprocal coordination during asynchronous bilateral activities (39). As a result, walking becomes less automated and requires greater top-down motor control. Within this context, our observed RPMC–motor performance correlations provide direct evidence that enhanced PMC engagement reflects increased motor planning demands in individuals with CP. Importantly, increased cortical activation should not be interpreted as inherently beneficial. Over-activation may represent adaptive support to maintain walking, but it may also reflect reduced neural efficiency or increased cortical “effort” due to limited automatic control. In line with this possibility, greater right premotor activation was associated with poorer motor performance (lower GMFM-E and slower 10-m walking speed), suggesting that children with lower functional capacity may require greater premotor resources for gait control.

Other explanations should also be considered. Increased prefrontal and premotor activation during walking may reflect reduced gait automaticity and greater reliance on top-down executive and motor planning resources, as commonly reported in fNIRS gait studies of clinical populations (40). However, over-activation may also indicate neural inefficiency or increased cortical effort, particularly when greater activation is associated with poorer performance (41). In addition, the capacity-limitation account suggests that increased activation may represent compensatory recruitment up to a resource ceiling, beyond which performance deteriorates, providing a useful framework for interpreting over-recruitment during gait (41). Finally, factors such as balance confidence/fear-related attentional load and methodological constraints inherent to walking-fNIRS should be acknowledged when interpreting frontal activation (31).

Additionally, no significant differences were observed in the bilateral SMC or visual cortex (VC). The absence of VC differences may reflect the relatively low visual demand of our paradigm, which involved straight walking at a self-selected pace under stable visual conditions, without externally imposed visual challenges (e.g., optic-flow perturbations or obstacle negotiation) (42). However, the absence of significant activation in the bilateral SMC does not align with our hypothesis and contradicts the previous study (20). Considering the anatomical location of the lower limb projections in the cortex, which are situated medially in the SMC, this area is almost beyond the detection limit of fNIRS (16). As a result, tasks that predominantly involve lower limb movements, such as walking, may show reduced detectable activation in the sensorimotor cortex when assessed using current fNIRS technology.

However, several limitations should be acknowledged. First, due to the limited channel number and spatial resolution of fNIRS, our ROIs did not cover certain gait-related cortical regions (e.g., superior parietal cortex), and the premotor subregions (PMA vs. SMA) could not be distinguished. In addition, fNIRS is restricted to superficial cortical layers and cannot assess subcortical structures essential for locomotor control, such as the basal ganglia and cerebellum; therefore, our findings should be interpreted as reflecting cortical-level involvement during walking rather than the entire locomotor network. Second, although larger than most previous reports, the sample size remains modest. To ensure diagnostic homogeneity and standardized testing, we restricted the cohort to children with spastic diplegic CP at GMFCS Levels I–II; thus, the results may not be generalizable to more severe CP (GMFCS III–V) or other subtypes. Third, because the study was cross-sectional, the functional meaning of increased prefrontal and premotor activation cannot be determined, and over-activation may reflect either compensatory recruitment or reduced neural efficiency. Finally, gait kinematics/EMG and orthotic conditions were not assessed, and between-group differences in preferred walking speed may have influenced cortical activation. Future studies with larger samples and multimodal designs (e.g., gait analysis, EMG, and longitudinal/interventional paradigms) are warranted.

## 5 Conclusion

In conclusion, significant differences in cortical activation patterns between children with spastic diplegic CP and CTD during walking are highlighted in this study. The over-activation in the right RPFC, LPFC, and RPMC in CP suggests a compensatory mechanism for motor planning and control. The critical role of the prefrontal and premotor cortices in managing motor deficits in children with spastic diplegic CP is underscored by these findings. These findings may help inform future research exploring interventions that target these cortical areas to improve gait and motor function in children with cerebral palsy.

## Data Availability

The raw data supporting the conclusions of this article will be made available by the authors, without undue reservation.
